# Preterm-Born Young Women Have Weaker Hand Grip Strength Compared to Their Full-Term-Born Peers

**DOI:** 10.3390/children10121898

**Published:** 2023-12-07

**Authors:** Ella Bruun, Pauli Pätsi, Markku Leskinen, Krista Björkman, Petri Kulmala, Mikko P. Tulppo, Marita Valkama, Marja Ojaniemi

**Affiliations:** 1 Department of Pediatrics, Oulu University Hospital, University of Oulu, Wellbeing Services County of North Ostrobothnia, 90220 Oulu, Finlandmarja.ojaniemi@oulu.fi (M.O.); 2 Research Unit of Clinical Medicine, University of Oulu, 90014 Oulu, Finland; 3 Medical Research Center, Oulu University Hospital, University of Oulu, Wellbeing Services County of North Ostrobothnia, 90014 Oulu, Finland; 4 Faculty of Medicine, University of Oulu, 90014 Oulu, Finland; 5 Research Unit of Biomedicine and Internal Medicine, University of Oulu, 90014 Oulu, Finland

**Keywords:** preterm birth, hand grip strength, vitamin D, muscle strength

## Abstract

Prematurity has been linked to lower muscular fitness and increased morbidity across the human lifespan. Hand grip strength is widely used as a measure of muscle strength. Previous studies have shown inconsistent results regarding the role of vitamin D in hand grip strength. Here, we investigated hand grip strength and the effects of a yearlong vitamin D supplementation in healthy preterm-born young adults. We recruited 38 young adults born preterm at either ≤32 weeks’ gestation or <34 weeks’ gestation and weighing <1500 g, as well as 39 gender- and age-matched controls, for this study. Anthropometric measurements, hand grip strengths, and vitamin D concentrations were recorded. These investigations were repeated after a yearlong vitamin D supplementation intervention. There was a significant difference in the age- and gender-specific hand grip strength ranks between the preterm- and full-term-born young adults: 57.9% and 30.7%, respectively, were below average (*p* = 0.009). In the preterm-born group, the females had significantly lower hand grip strengths compared to their full-term-born peers, with a mean difference of −3.46 kg (95% CI: −6.68 to −0.247; *p* = 0.035). In a linear regression analysis, the preterm-born female adult height was negatively associated with hand grip strength (R^2^ = 0.24, F (1.43) = 13.61, *p* < 0.001). The vitamin D concentrations were increased after the supplementation period, with no association with hand grip strength. According to our results, preterm-born young females are at risk for lower muscle strength, independent of their current vitamin D status.

## 1. Introduction

The estimated rate of preterm birth is 7.9% of live births in Europe and 9.9% globally, with no change from 2010 to 2020 [1]. The survival rates after preterm birth increased from the 1980s to the 1990s with the implementation of modern treatments and surfactant availability [2]. However, increased mortality [3], morbidity, and long-term health consequences related to preterm birth are common in adulthood [4]. Preterm birth appears as a chronic condition in which morbidities linked to ageing appear earlier than in the full-term-born population [5].

Low muscular fitness has been associated with preterm birth [6], and muscle weakness is a feature of vitamin D deficiency. However, studies investigating the effects of vitamin D on muscle strength, especially in low S-25-OHD concentrations, have shown inconsistent results [7,8,9,10,11]. Vitamin D deficiency, measured as serum-25-hydroxyvitamin D (S-25-OHD) <50 nmol/L (<20 ng/mL), and insufficiency, measured as <72.5 nmol/L (<30 ng/mL), are recognised global health problems [12], though blood concentrations of S-25-OHD defining vitamin D deficiency or insufficiency remain controversial. Vitamin D receptors are found in the skeletal muscle tissue, and vitamin D has a role in skeletal muscle growth, muscle fibre size, and muscle cell repair, though the precise mechanisms thereof are still unclear [13,14]. Adequate vitamin D status seems to protect against a wide spectrum of disorders [15]. 

The hand grip test is widely used as a measure of muscle strength and has become an outcome marker of nutritional status [16]. Poor nutrition and vitamin D deficiency have been linked to weaker hand grip strength [16,17]. High protein and energy supplementation in malnutrition cases and vitamin D supplementation in vitamin D deficiency cases have been shown to increase hand grip strength [18,19]. In addition, low birth weight has been associated with weaker hand grip strength in early adulthood [20,21]. Early preterm birth at <34 weeks’ gestation has been associated with lower hand grip strength, but the difference between preterm and full-term birth is attenuated when an adjustment is made for birth weight [6].

Here, we investigated whether preterm birth is associated with weaker hand grip strength and whether there are differences between genders. We also assessed the effects of vitamin D levels on hand grip strength in healthy preterm-born young adults compared to their healthy peers born at term. Finally, we investigated the effects of a yearlong vitamin D supplementation with a S-25-OHD target level of 80–120 nmol/L on hand grip strength in the preterm-born subjects.

## 2. Materials and Methods

**Figure 1:**
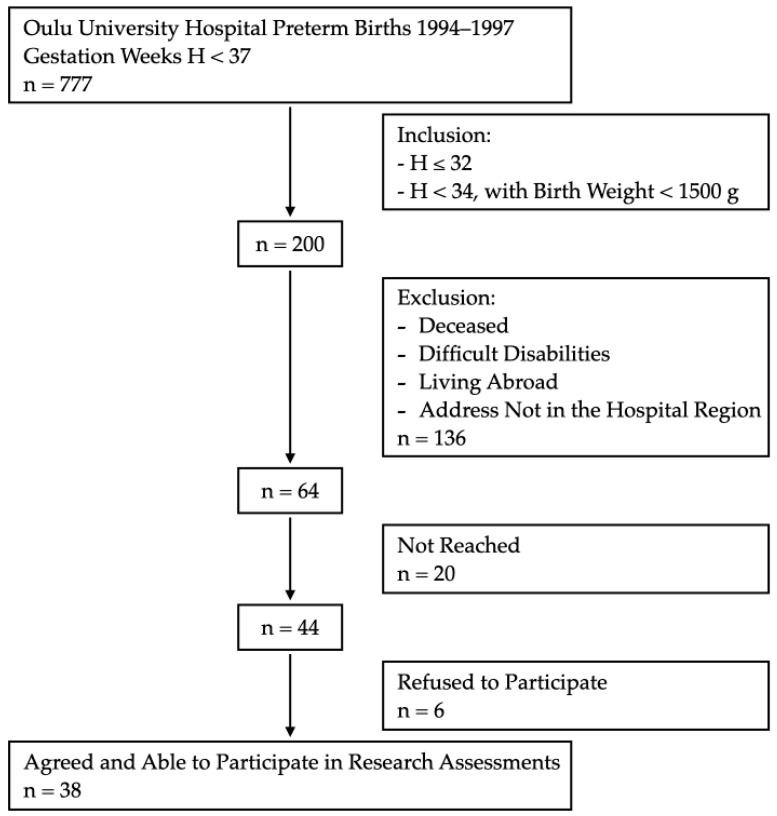
Flow chart of preterm-born participant recruitment.

The preterm-born participants for this study were selected from infants treated at the Oulu University Hospital between 1994 and 1997. These participants were identified using hospital records. Healthy young adults born either before 34 weeks’ gestation with very low birth weight (VLBW, <1500 g) or at ≤32 weeks’ gestation met the inclusion criteria. From this population, those who were deceased; living abroad or outside the tertiary hospital district; and with complex physical, intellectual, or social disabilities that would have prevented the planned investigations were excluded from this study. A more detailed flow chart of the participant recruitment is presented in Figure 1.

Birth weight, birth length, and gestational week data were gathered from the medical databases of the hospital. Based on the International Statistical Classification of Diseases and Related Health Problems (ICD-10), participants born with birth weights of <1000 g were classified into an extremely low birth weight (ELBW) subgroup. Participants with birth weights of <2 SD below average for their gestational ages (P05.0), birth weights and lengths of <2 SD below average for their gestational ages (P05.1), and birth lengths of <2 SD below average for their gestational ages (P05.9) were classified into a small for gestational age (SGA) subgroup. The age- and gender-matched full-term-born control participants were recruited from the population of healthy young adults living in the tertiary hospital district. Some had been recruited for an earlier study in childhood as controls and agreed to participate again. Other controls were recruited from among medical or nursing students or their peers outside of the health care setting. The birth data of the controls were gathered using questionnaires.

The power calculation for the number of participants in this study was performed by estimating that an inadequate vitamin D status (S-25-OHD < 50 nmol/L) would be found in 23.5% of adult participants. This percentage was based on a report showing the prevalence of vitamin D inadequacy in Northern Finland during the years 2012–2013 [22]. With the aim of decreasing the percentage of the population with inadequate vitamin D status from 24% to 2.4% with vitamin D supplementation, the minimum number of participants required was calculated to be 35 per group. To reduce the effects of losses, the number recruited for the intervention was 38 per group.

Height, weight, and waist and hip circumference for all participants were measured by one investigator (E.B.). Height (cm, to an accuracy of 0.1 cm) was measured using a digital standing stadiometer (Seca gmbh, Hamburg, Germany; mod: 2641900099). Weight (kg, to an accuracy of 0.1 kg) was measured using a digital flat scale (Seca Delta model 707, Seca gmbh, Hamburg, Germany; mod: 3093309), with participants wearing light indoor clothing. The waist and hip circumferences (cm, to an accuracy of 0.1 cm) were measured using a soft measuring tape. The waist-to-hip ratio was calculated as the waist circumference (cm) divided by the hip circumference (cm). The body mass index (BMI, kg/m^2^) was calculated as the measured weight (kg) divided by the measured height (m) squared.

Muscle mass was measured for the preterm-born participants, as part of the body composition measurements, using dual X-ray absorptiometry (DXA, GE Lunar iDXA, GE HealthCare, Madison, WI, USA, 2015).

All laboratory samples, from all participants, were drawn by a single investigator (E.B.). Serum 25-hydroxyvitamin D concentration (S-25-OHD, nmol/L) was measured using an immunochemiluminometric method, and plasma calcium (P-Ca, mmol/L), plasma phosphorus (P-Pi, mmol/L), and plasma alkaline phosphatase (AFOS, U/L) were measured using a photometric method. All laboratory samples were analysed in the Northern Finland Laboratory Centre (NordLab, Oulu, Finland). NordLab is a testing laboratory (SFS-EN ISO 15189:2013) accredited by the Finnish Accreditation Service (FINAS, Helsinki, Finland).

Hand grip strength was measured from all participants. Measurements were made using a SAEHAN hydraulic hand dynamometer (SAEHAN corporation, Changwon, Republic of Korea). All participants were assessed in the sitting position, straight-backed, with feet on the floor, the elbow flexed at 90 degrees, and the forearm and wrist in a neutral position. Maximal hand grip strength was measured three times for each hand, with 3–5 s per measurement. The participants were allowed to switch hands between measurements. The best result for each hand was recorded, as well as the age- and gender-specific ranking from 1 through 5 (1 = markedly below average, 2 = somewhat below average, 3 = average, 4 = somewhat above average, 5 = markedly above average), based on the Finnish national health study results from 2017 [23,24]. Handedness was asked from each participant prior to the hand grip test.

For each participant, we assessed the average protein intake in grams divided by the measured weight (kg) using a food diary that spanned five days. All food diaries were analysed by a single investigator (E.B.) using the national Food Composition Database in Finland (Fineli), which is maintained by the Finnish Institute for Health and Welfare [25]. Exercise habits were assessed via a questionnaire, with an aim to assess whether the participants fulfilled the WHO guidelines for physical activity [26]. 

To investigate the effects of the yearlong vitamin D supplementation on hand grip strength in these preterm-born young adults, S-25-OHD concentrations were measured on the first visit, every 4 to 6 months during the intervention to assure safe concentration, and on the control visit after a year of supplementation. The target concentration for S-25-OHD was 80–120 nmol/L. The amount of vitamin D supplementation was not increased based on the control values during the intervention period, except for the participants whose supplementation was started based on S-25-OHD <80 nmol/L in the 6-month control. The supplementation protocol based on the S-25-OHD concentration is presented in Table 1.

All investigations were repeated for the preterm-born participants after the supplementation period. 

Statistical analyses were performed using SPSS version 28.0 (IBM SPSS Statistics, IBM Corporation, Armonk, NY, USA). Herein, normally distributed data are reported as means with standard deviations (SD), and non-normally distributed data are also reported with medians and percentiles. Comparisons between the study groups were made with an independent-samples *t*-test and a Mann–Whitney U test for the normally and non-normally distributed variables, respectively. Between the first and control visits, comparisons for the preterm-born group were made with a paired-samples *t*-test and a Wilcoxon signed-ranks test for the normally and non-normally distributed variables, respectively. Comparisons of the grip strength ranks between the groups and between the first and control visits for the preterm group were made with a χ^2^ test and a Wilcoxon signed-ranks test, respectively. The level of statistical significance was set at *p* < 0.05.

This study was conducted in accordance with the Declaration of Helsinki and approved by the Ethics Committee of Oulu University Hospital (ET 92/2017). Written informed consent was obtained from all participants prior to these investigations. This study is part of a larger research project: ClinicalTrials.gov NCT04342078. 

## 3. Results

### 3.1. Study Participants

A total of 38 preterm-born young adults out of the 64 eligible subjects (59.4%) born in the years 1994–1997 participated in this study. The group consisted of 22 females (58%) and 16 males (42%). The age- and gender-matched full-term-born control group consisted of 23 females (59%) and 16 males (41%). One female control was unable to participate in the investigations in another study of a larger project, for which another control was recruited, and both were included in this study. One male from the preterm-born group withdrew from this study after the first visit.

Altogether, eight participants (21.1%), six female and two male, fulfilled the criteria for being born SGA; two were light (ICD-10 P05.0), three were short and light (P05.1), and three were short (P05.9) for their gestational ages. Six out of the thirty-eight participants (15.8%) were born with ELBWs of < 1000 g. The gestational ages of the preterm-born group varied between 27 and 32.9 weeks, with a mean of 30.5 gestational weeks. The mean birth weights, birth lengths, and gestational ages of the study participants are shown in Table 2.

The preterm-born females were significantly shorter than the corresponding controls, with a mean difference in height of 4.0 cm (95% CI: −7.2 to −0.8; *p* = 0.016). There were no differences in height between the preterm-born females born SGA and those born at appropriate sizes for their gestational ages (95% CI: −8.1 to 2.9; *p* = 0.338). The preterm-born participants were younger than their full-term-born controls, with a mean age difference of 0.7 years (95% CI: −1.0 to −0.3). There were no statistically significant age differences between the groups of males, but the preterm-born females were younger than their full-term-born controls, with a mean age difference of 1.1 years (95% CI: −1.5 to −0.7). There were no significant weight, BMI, or waist-to-hip ratio differences between the groups of females or those of males. More detailed birth and present characteristics are presented in Table 2.

### 3.2. Vitamin D

At the beginning of this study, the mean vitamin D concentration for the preterm-born group was 60.3 (SD 18.7) nmol/L. Nine participants (23.7%) in this group had S-25-OHD < 50 nmol/L, and seven (18.4%) had S-25-OHD in the target range of 80–120 nmol/L. In the control group, the mean concentration of S-25-OHD was 57.9 (14.9) nmol/L. Ten participants (25.6%) had S-25-OHD < 50 nmol/L, and four (10.3%) had vitamin D levels in the target range. There was no significant difference in vitamin D levels between the preterm and full-term study groups or between the female and male participants.

On the first visit, the mean vitamin D concentration was 59.2 (17.1) nmol/L during the dark season (November–May) and 58.9 (16.5) nmol/L during the light season (June–October). Most of the laboratory values obtained during this study were measured during the dark season, with 64.9% at the first visit and 83.8% at the control visit. There were no significant differences between the preterm-born and control groups in the timing of the laboratory measurements.

The detailed results of the serum 25-hydroxyvitamin D (S-25-OHD, nmol/L), plasma calcium (P-Ca, mmol/L), plasma phosphorus (P-Pi, mmol/L), and plasma alkaline phosphatase (AFOS, U/L) concentrations are presented in Table 3. 

### 3.3. Hand Grip Strength

In total, 30 (78.9%) adults in the preterm-born group and 37 (94.9%) in the control group reported themselves to be right-handed (χ^2^-test, *p* = 0.038). Eight (21.1%) in the preterm group and nine (23.1%) in the control group had higher hand grip strength in the non-dominant hand, with no differences between the groups. Six adults each in the preterm-born (15.8%) and control groups (15.4%) had equal grip strength between hands.

In the preterm-born group, females had significantly lower hand grip strengths compared to their full-term-born peers, with a mean difference of −3.46 kg (95% CI: −6.68 to −0.247; *p* = 0.035), as is shown in Table 4. There were also significant differences in the age-and gender-specific hand grip strength ranks, with 50% of the preterm-born females ranking below average as compared to 13% of their full-term-born peers (χ^2^-test, *p* = 0.029). Linear regression was used to test if the preterm-born females’ shorter adult height was associated with their hand grip strength. The overall regression was statistically significant (R^2^ = 0.24, F (1.43) = 13.61, *p* < 0.001). It was found that every 1 cm increase in height increased grip strength by 0.484 kg (95% CI: 0.219 to 0.748) in young adult females. Birth weight and height correlated positively with hand grip strength in the females (r_s_ = 0.33, *p* = 0.027 and r_s_ = 0.32, *p* = 0.036, respectively), but gestational age did not. Neither age, nor weight, nor BMI correlated significantly with hand grip strength in females at the time of these investigations.

There were no significant differences in hand grip strength or rank between the groups of males. Neither age, nor weight, nor height correlated significantly with hand grip strength in males at the time of these investigations, but there was a borderline correlation with BMI. We used linear regression to test whether BMI could significantly predict hand grip strength. The overall regression was borderline significant (R^2^ = 0.12, F(1,30) = 4.19, *p* = 0.05). It was found that for every 1 kg/m^2^ increase in BMI, grip strength increased by 0.697 kg (95% CI: 0.001 to 1.393). In the males, there was no correlation between birth weight, birth height, or gestational weeks and hand grip strength.

In the preterm-born females, birth weight, but not birth height or gestational age at birth, correlated positively with absolute muscle mass in adulthood (r_p_ = 0.44, *p* = 0.042), which, in turn, correlated with the best hand grip strength (r_p_ = 0.65, *p* < 0.001). There were no correlations between birth characteristics, muscle mass, and hand grip strength in preterm-born males or the whole preterm-born group.

**Figure 2:**
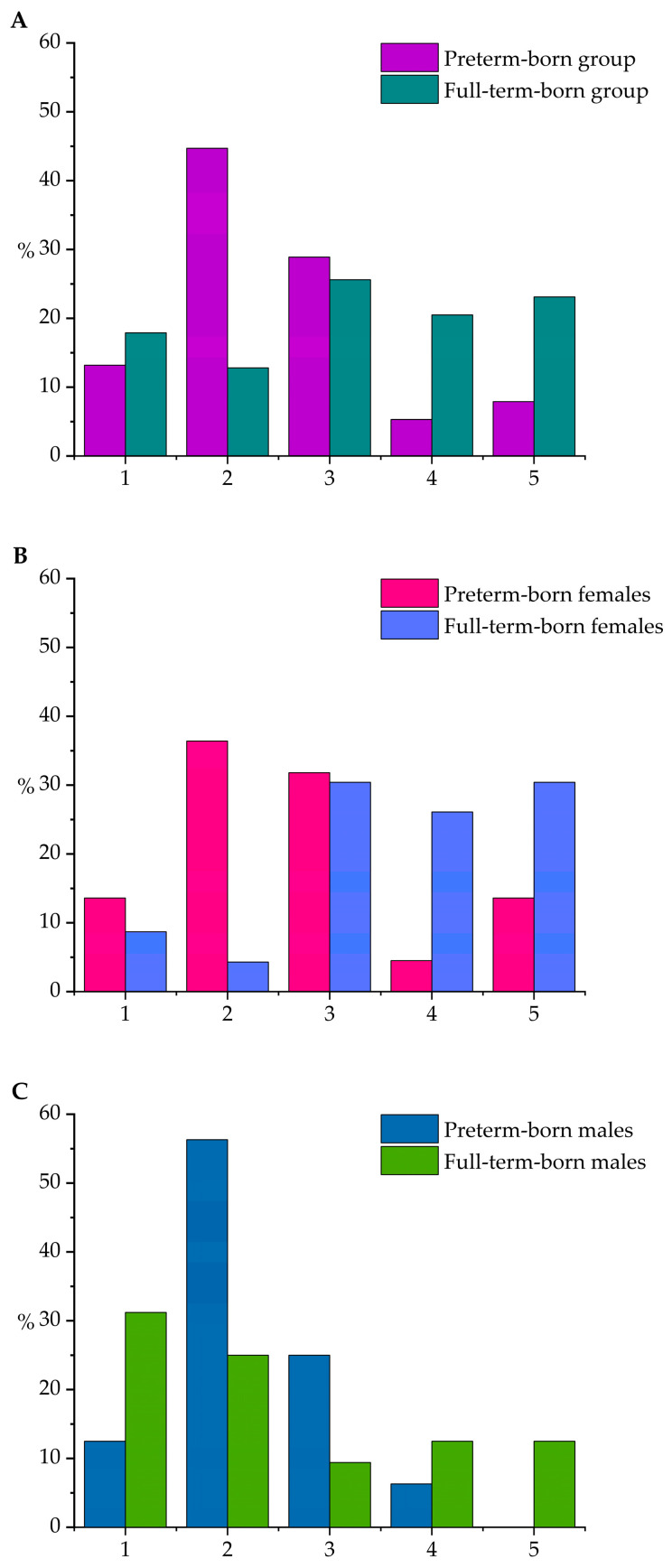
Comparisons in hand grip strength rank between preterm- and full-term-born study groups (**A**), females (**B**), and males (**C**). Hand grip strength was ranked as follows, based on the Finnish national health study results of 2017: 1 = markedly below average, 2 = somewhat below average, 3 = average, 4 = somewhat above average, 5 = markedly above average [23,24].

There were significant differences in the age- and gender-specific hand grip strength ranks between the preterm-born and full-term-born young adults, at 57.9% and 30.7% below average, respectively (χ^2^-test, *p* = 0.009). The hand grip strength ranks between the groups are presented in Figure 2. There were no significant differences in rank between all of the males and females, the preterm-born males and females, or the full-term-born males and females.

### 3.4. Protein Intake and Exercise Habits

In the preterm group, 35 (92.1%) returned the food diaries and the exercise questionnaires, and in the control group, 36 (92.3%) returned the food diaries and 35 (89.7%) returned the exercise questionnaires. The full-term-born group had a slightly higher protein intake compared to the preterm group, with a mean difference of 0.19 g/kg (95% CI: 0.03 to 0.35; *p* = 0.018). The full-term-born males also had a higher protein intake compared to the preterm-born males, with a mean difference of 0.28 g/kg (95% CI: 0.02 to 0.55; *p* = 0.037). There was no difference in protein intake between the groups of females. In the preterm-born group, 22 (62.9%) fulfilled the WHO recommendations for aerobic physical activity, which is significantly less compared to the 31 (88.6%) in the control group (χ^2^-test, *p* = 0.012). There were no differences between the groups in strength training, with 17 (48.6%) and 20 (57.1%) fulfilling the WHO recommendations in the preterm and control groups, respectively. The protein intake of each group and comparisons between the groups in aerobic exercise and strength training can be seen in Table 5.

### 3.5. Vitamin D and Hand Grip Strength

During the intervention, the mean S-25-OHD concentration increased significantly, from 60.3 (18.7) nmol/L to 82.9 (25.8) nmol/L, with a mean difference of 22.8 nmol/L (95% CI: 13.6 to 32.0; *p* < 0.001). 

There was no significant change in hand grip strength or rank after the yearlong intervention period. There was no correlation between S-25-OHD concentration and hand grip strength in either group. S-25-OHD did not correlate with weight, height, or BMI either.

## 4. Discussion

In our study, preterm-born young females had significantly lower hand grip strengths compared to their full-term-born peers. Of all the preterm-born participants, 57.9% had hand grip strengths below average when compared with national age- and gender-specific reference values [23,24], which was also significant in comparison to their full-term-born controls. Lower grip strength has previously been found in adults born with ELBW [27]. Weak grip strength in preterm-born young adults is a worrisome finding, since grip strength has previously been inversely associated with multiple chronic diseases [28]; coronary heart disease; stroke [29]; and increased cardiovascular, non-cardiovascular, and overall mortality [30]. 

The mechanisms of how prematurity affects muscular fitness are unclear. Low birth weight in full-term-born individuals has been found to affect skeletal muscle morphology towards a dysmetabolic phenotype, including reduced insulin sensitivity [31], which is a possible mechanism for reduced grip strength and increased morbidity and mortality. Similar findings have been made in a rat model of preterm birth, in which morphological changes in skeletal muscle were characterised by chronic inflammation and the alterations were maintained into adulthood [32]. Still, the precise molecular mechanisms thereof need further study. 

In the current study, preterm-born young adult females were significantly shorter than their full-term-born peers. In line with our findings, a previous study has reported similar results; decreased gestational age was associated with 1.1 mm lower adult height in females for every week of earlier birth [33]. However, studies that have associated birth size with adult height in males found birth length and weight relative to gestational age to have more influence on adult height than gestational age alone [34,35]. A recently published Brazilian cohort study found preterm-born males to have shorter adult heights compared to their full-term-born peers, but no such difference in females [36]. A British study found no difference in adult height between preterm- and full-term-born infants with appropriate weights for their gestational ages [37]. On the other hand, extremely preterm birth, before 26 weeks of gestation, seems to compromise adult height in comparison to full-term birth, with no difference between genders [38]. Current evidence suggests a possible gender difference, though study designs that have used low birth weight and low gestational age variably have offered conflicting results. Preterm-born females seem to have a greater risk for smaller adult size and, thus, weaker hand grip strength than preterm-born males. Our preterm-born females were statistically younger than their controls; however, when full adult height was attained, we interpreted that the mean age difference of 1.1 years had no significance in our study. Supporting normal growth and normal motor development as well as encouraging physical activity in preterm-born children are important to ensure muscle strength in adulthood, since both adult size and physical activity affect both muscle mass and grip strength. There is a need to increase the awareness of health care professionals working in children’s and school health about the risks involved with being born preterm so they can inform and support families as their children grow.

There was more left-handedness in our preterm-born group than in the full-term-born controls. Preterm birth has been associated with increased non-right-handedness. The mechanisms proposed include birth stress, early brain injury, and genetic factors, but there is still a lack of sufficient evidence to support these theories [39].

Vitamin D deficiency has previously been associated with reduced hand grip strength [17]. We found no differences in the vitamin D levels between the study groups. The yearlong vitamin D supplementation raised the vitamin D levels in the preterm-born participants, with no effect on hand grip strength. The previously reported positive effects of vitamin D on hand grip strength were for individuals with vitamin D deficiency (S-25-OHD < 25–50 nmol/L) [17,19]. We had no participants with S-25-OHD concentrations of <25 nmol/L in our study. We realise that one year is a short follow-up for changes in muscle strength. If our study population had vitamin D deficiency, the possible effects of the vitamin might have been seen within a relatively short period of time, as in the study by Kalliokoski et al. [19], where a positive effect on grip strength was seen within 10 months. Whether a longer vitamin D supplementation would influence grip strength without a noted deficiency remains open.

We found a correlation between birth weight and absolute muscle mass in the preterm young adult females, which also correlated with their maximal hand grip strength, but there was no such finding in the males. In a previous study, adolescents at 11 years of age born with ELBW had lower heights, weights, and lean body masses compared to their peers born at term. Catch-up growth in weight in early childhood seems to be beneficial in terms of body composition in former ELBW children [40]. In a study conducted on preterm-born children at 7 years of age, the observed group had lower heights, weights, and maximal grip strengths compared to the controls. Higher birth weight was positively associated with higher weight, height, and grip strength at 7 years [41]. It seems that preterm-born individuals are already at risk of smaller body sizes and lower lean masses in childhood and young adolescence, which could be implied by their lower hand grip strength. In the treadmill stress test of another study of extremely preterm-born young adults of 18 years, those participants had modestly reduced exercise capacities. The preterm group reported exercising less than their peers in their free time, but there was a positive association between reported exercise habits and exercise capacity, supporting the positive implications of exercise in preterm-born young adults [42].

One of the strengths of our study is that the population consisted of young adults of Finnish nationality, making the population genetically homogenic. We used relatively new national reference data for gender- and age-specific hand grip strength, which applied well to our study population. There is seasonal variation in vitamin D concentrations in countries with insufficient food fortification with this vitamin, especially in the Nordic latitudes [22,43,44]. The Finnish vitamin D food fortification programme and national recommendations have reduced seasonal variation in Northern Finland [22], but we still took that variation into account in our study. We are aware that although vitamin D concentration had no association with hand grip strength, these results must be interpreted with caution due to the relatively short intervention period. 

There are several sources of bias in our study. First, we gathered data on recent nutritional and exercise habits to give us approximates of protein intake and amounts of exercise, but these data were self-reported by the participants, and the reports were variable in quality. Second, there are many factors that we could not standardise in a study setting, such as exercise habits, diet, and other lifestyle choices; extracurricular activities; and occupation, and we cannot exclude their impact on our study results. These same factors, as well as genetics, also have roles earlier in childhood and adolescence. Third, our control group was partly recruited from students in a health care setting, which may have caused bias towards a healthier lifestyle and thus affected our study results in favour of the control group. A weakness in our study is that body composition by DXA was only measured in the preterm-born participants, so we were unable to compare their muscle mass data with those of their full-term-born peers. 

## 5. Conclusions

Preterm-born females are at a risk for reduced muscle strength in young adulthood. The mechanisms thereof are unclear, but shorter adult stature may play a role. Here, vitamin D concentration had no association with hand grip strength, although these results must be interpreted with caution due to the relatively short intervention period. As both preterm birth and decreased hand grip strength have been associated with increased morbidity and mortality, the interaction between these factors warrants further research. The finding that prematurity poses a risk for weak muscle strength already at young adulthood highlights the importance of maintaining physical fitness and muscle strength in this population throughout one’s lifespan.

## Figures and Tables

**Table 1:** Daily vitamin D supplementation based on S-25OHD concentration.

Serum-25-OHD Concentration	Daily Vitamin D Supplementation
<30 nmol/L (<12 ng/mL)	4000 IU = 100 µg
30–50 nmol/L (12–20 ng/mL)	2000 IU = 50 µg
50–79 nmol/L (20–31.6 ng/mL)	1000 IU = 25 µg
≥80 nmol/L (≥32 ng/mL)	No additional supplement

**Table 2:** Birth and present characteristics of preterm-born participants and their full-term-born controls on the first visit of this study.

Birth Characteristics	PT (*N* = 38)	FT (*N* = 39)	*p*-Value
Birth Weight, Grams, Mean (SD)	1397.0 (300.7)	3517.2 (470.0)	<0.001
Birth Length, Centimetres, Mean (SD)	39.2 (2.5)	50.2 (1.9)	<0.001
Gestational Age at Birth, Weeks, Mean (SD)	30.5 (1.3)	39.2 (1.1)	<0.001
SGA (P05.0, P05.1, or P05.9), Number (%)	8 (21.1)	1 (2.6)	
ELBW, Number (%)	6 (15.8)	-	
Female/Male, Number	22/16	23/16	
**Present Characteristics**			
Age, Years, Mean (SD)	23.6 (0.7)	24.3 (0.8)	<0.001
Female	23.3 (0.6)	24.4 (0.8)	<0.001
Male	24.0 (0.7)	24.1 (0.8)	0.836
Weight, Kilograms, Mean (SD) Female			
	62.4 (11.4)	63.9 (13.4)	0.725
		* 60.2 (57, 65.5)	
Male	72.5 (10.4)	77.6 (12.4)	0.218
Height, Centimetres, Mean (SD)			
Female	162.6 (5.5)	166.7 (5.2)	0.016
Male	176.2 (5.9)	179.2 (7.2)	0.222
Waist-to-Hip Ratio, Mean (SD)			
Female	0.78 (0.06)	0.76 (0.04)	0.278
Male	0.86 (0.05)	0.86 (0.04)	0.838
	* 0.86 (0.8, 0.9)		
BMI, kg/m^2^, Mean (SD)			
Female	23.5 (3.8)	22.9 (4.2)	0.440
		* 22.1 (21.0, 24.2)	
Male	23.3 (3.2)	24.1 (3.5)	0.509

* Median (25th, 75th percentiles) for non-normally distributed data. PT = preterm-born group, FT = full-term-born group, SGA = small for gestational age, ELBW = extremely low birth weight, BMI = body mass index (weight/height^2^).

**Table 3:** Serum-25-OHD concentrations with related parameters.

	PT First Visit	PT Control Visit	FT
S-25-OHD, nmol/L, Mean (SD)			
Female	61.6 (21.2) ^1^	81.4 (25.7) ^1^	56.9 (12.9)
Male	58.4 (15.0) ^1^	85.2 (26.5) ^1^	59.4 (17.7)
		* 79.0 (68.0, 93.0)	
All	60.3 (18.7) ^1^	82.9 (25.8) ^1^	57.9 (14.9)
		* 79.0 (63.5, 95.5)	
P-Ca, mmol/L, Mean (SD)			
Female	2.33 (0.08)	2.36 (0.08)	2.34 (0.09)
Male	2.38 (0.07)	2.39 (0.07)	2.34 (0.07)
All	2.35 (0.08)	2.37 (0.08)	2.34 (0.08)
P-Pi, mmol/L, Mean (SD)			
Female	1.10 (0.19)	1.18 (0.18)	1.20 (0.20)
Male	1.04 (0.16) ^1^	1.17 (0.18) ^1^	1.14 (0.17)
	* 1.04 (0.92, 1.09)		
All	1.07 (0.18) ^1,2^	1.17 (0.18) ^1^	1.18 (0.19) ^2^
P-AFOS, U/L, Mean (SD)			
Female	62.8 (13.9)	60.1 (16.8)	61.7 (18.8)
		* 56.0 (48.5, 70.0)	* 57.0 (48.0, 70.0)
Male	70.6 (12.0)	67.2 (12.0)	67.0 (21.1)
			* 61.5 (51.3, 79.5)
All	66.1 (13.5) ^1^	63.1 (15.2) ^1^	63.9 (19.7)
			* 61.0 (50.0, 71.0)

* Median (25th, 75th percentiles) for non-normally distributed data; ^1^ significant difference between PT at first and control visits, with *p* < 0.05; ^2^ significant difference between PT at first visit and FT, with *p* < 0.05. PT = preterm-born group, FT = full-term-born control group.

**Table 4:** Best hand grip strength results based on gender in preterm- and full-term-born young adults.

	PT First Visit	PT Control Visit	FT
	*N* = 38, 22 F/16 M	*N* = 38, 22 F/15 M	*N* = 39, 23 F/16 M
**Best Hand Grip, kg (SD)**			
Female	30.3 (5.9) ^1^	30.2 (4.8)	33.8 (4.8) ^1^
Male	47.4 (5.4)	49.6 (4.6)	49.3 (7.8)

^1^ Significant difference between PT at first visit and FT, with *p* < 0.05. PT = preterm-born group, FT = full-term-born control group.

**Table 5:** Confounding factors that affected hand grip strength.

	PT	FT	*p*-Value
**Protein Intake (g/kg) *n* = 71**	1.12 (0.38)	1.31 (0.28)	0.018
Female, *n* = 43	1.13 (0.38)	1.27 (0.28)	0.190
Male, *n* = 28	1.10 (0.39)	1.38 (0.28)	0.037
**Fulfil WHO Recommendation, *n* = 70**			
Aerobic Exercise, Number (%)	22 (62.9)	31 (88.6%)	0.012
Female, *n* = 42	12 (60%)	20 (90.9%)	0.019
Male, *n* = 28	10 (66.7%)	11 (84.6%)	0.274
Strength Training, Number (%)	17 (48.6%)	20 (57.1%)	0.473
Female, *n* = 42	9 (45%)	12 (54.5%)	0.537
Male, *n* = 28	8 (53.3%)	8 (61.5%)	0.662

PT = preterm-born group, FT = full-term-born control group.

## Data Availability

The data presented in this study are available on request from the corresponding authors. The data are not publicly available due to privacy.
